# Telemedicine Patient Satisfaction Across Nine Countries: A Cross-Sectional Comparative Study

**DOI:** 10.3390/healthcare14203493

**Published:** 2026-10-17

**Authors:** Andrew N. Mason, Matt Brown, Kevin Mason

**Affiliations:** 1 Faculty of Medicine, Juntendo University, 2-1-1 Hongo, Bunkyo, Tokyo 113-8421, Japan; 2 School of Business-Data Analytics, Arkansas Tech University, 106 West O Street, Russellville, AR 72801, USA; hbrown11@atu.edu; 3 School of Business-Marketing, Arkansas Tech University, 106 West O Street, Russellville, AR 72801, USA; kmason@atu.edu

**Keywords:** telemedicine, telehealth, patient satisfaction, satisfaction dimensions, multicountry patient satisfaction comparisons

## Abstract

**Background/Objectives:** This study examined telemedicine patient satisfaction as a multidimensional construct. In addition to overall satisfaction, four satisfaction dimensions were examined (health benefits, patient-centered care, financial costs, and non-financial costs). The aim of this study was to determine if overall satisfaction and the predictive capacity of the defined satisfaction dimensions varied across the nine countries studied. **Methods:** Subjects were telemedicine patients from Australia, Brazil, Canada, Germany, India, Japan, Mexico, the United Kingdom, and the United States. A questionnaire using Likert scale items measured the patient satisfaction variables. Telemedicine patient overall satisfaction was the dependent variable, and the four dimensions of satisfaction were the independent variables. ANOVA tested for differences in patient overall satisfaction. Regression analysis examined the significance of the four satisfaction dimensions as predictors of overall satisfaction. Random forest machine learning assessed which dimension in each country was the best predictor of overall satisfaction. **Results:** Telemedicine patient satisfaction was found to differ across countries. Patients in India and Brazil had the highest mean overall satisfaction, whereas patients in Japan had the lowest. Patient-centered care was the most important predictor of overall satisfaction in all countries except the United States and Japan, where the greatest predictor was telemedicine non-financial costs. **Conclusions:** Telemedicine patient satisfaction differences were observed across the countries studied. Telemedicine service providers may need to adapt their service offerings to align with local patient expectations and norms to maximize satisfaction. A multidimensional examination of telemedicine patient satisfaction may be able to provide potential insights into target areas for service improvements.

## 1. Introduction

In a world of rapidly advancing technology, telemedicine has revolutionized the healthcare industry. Over the last decade, the expansion of telemedicine services has reshaped the way patients access healthcare services. Telemedicine has surged in popularity worldwide for many reasons, such as greater healthcare accessibility to underserved communities, more convenience to patients, and infection risk reduction for patients and healthcare providers [1,2,3].

Patients’ satisfaction with telemedicine is a function of their perceptions and the importance they place on various aspects of the service. Across countries, it is conceivable that patients’ experiences and their perceptions of those experiences may vary. Factors that may differ across and within countries that could impact patient experience, and thusly satisfaction, with telemedicine include deviations in telemedicine cost/reimbursement [4], technology infrastructure [5], patient access [6,7,8], telemedicine policies [9], healthcare provider technology training [10,11], patient demographic profile [4,12], and culture [13].

Understanding telemedicine patient satisfaction within a given country is essential because the identification of factors that drive patient overall satisfaction for a given country can serve as a basis for quality improvement initiatives. Unfortunately, systematic literature reviews of measures used for telemedicine patient satisfaction have found a lack of uniformity in measurement methods, as various dimensions of telemedicine patient satisfaction have been used to assess it over the years [14,15].

To address this gap, Mason [16] conducted multidimensional research on telemedicine patient satisfaction using 32 satisfaction measures. Mason [16] used confirmatory factor analysis to validate the questionnaire, and the measurement items revealed four primary predictor dimensions of overall satisfaction. Mason [16] defined the dimensions as health benefits, patient-centered care, financial costs, and non-financial costs. The health benefits dimension consisted of patient perceptions of healthcare aspects such as access, safety, and the achievement of health outcomes. The telemedicine patient-centered care dimension was measured with patient perceptions of the provider’s interpersonal relationship skills, such as the provider’s responsiveness, attentiveness, and trustworthiness. Telemedicine financial costs included various monetary costs associated with the telemedicine service received. Telemedicine non-financial costs included time-saving conveniences and service complexities such as technology problems. This study utilized the measures identified by Mason [16] to perform an exploratory, multidimensional examination of telemedicine satisfaction across multiple countries. The purpose of this study is to explore the following research questions:

R_1_: Does telemedicine patient *overall satisfaction* vary across countries?

R_2_: Does the most important satisfaction dimension for predicting overall satisfaction vary across countries?

## 2. Methods

A cross-sectional, multicountry, online survey was conducted via convenience sampling by Centiment (Denver, CO, USA), a marketing research company, which has telemedicine patient panels in various nations. Centiment offered five U.S. dollars as compensation for survey respondents’ time and reached as broad and representative an audience as possible using many outlets. Primary recruitment resources include social media sites, such as Facebook and LinkedIn. Centiment’s security utilizes technology that combines IP address, device type, screen size, and cookies to ensure only unique panelists participate in the survey. Centiment defends against bots using invisible ReCaptcha. Upon qualifying for the survey, Centiment unlocked the survey within the respondent panel’s dashboard, allowing the respondent to enter. To drive participation in the survey, Centiment ran batches of notifications to targeted groups of respondents. Notifications included a mix of email and push notifications based on respondent preferences.

Data was collected in 2024, January 5 to February 13, from subjects who received telemedicine services within the previous 12-month period and were at least 20 years of age or older at the time of taking the questionnaire. Subjects were not asked to share personally identifiable information; thus, they remained anonymous. To prevent duplication of responses, a custom variable was assigned to each subject. Due to budget restrictions, language barriers, and the availability of telemedicine patient panel access, data was collected from patients representing all continents except Africa and Antarctica. The country panels used were from Australia, Brazil, Canada, Germany, India, Japan, Mexico, the United Kingdom (U.K.), and the United States (U.S.). Thus, countries from five continents were represented.

An English version of Mason’s [16] questionnaire was used to collect data in Australia, Canada, India, the U.K., and the U.S. (see Supplementary Material S1). The questionnaire was translated to native languages in Brazil, Germany, Japan, and Mexico. To ensure context accuracy of the questionnaire, Centiment utilized language experts to oversee the process of translating the English version of the questionnaire into other languages using Google Translate and then back-translating into English. A total of 1950 patients participated with the following sample sizes:
Australia—217Brazil—234Canada—206Germany—204India—234Japan—213Mexico—217U.K.—208U.S.—217

The dependent variable (DV) was telemedicine patient overall satisfaction*,* and perceptions of the four dimensions of satisfaction were treated as the independent variables (IVs). More specifically, the IVs were patient perceptions of the health benefits, patient-centered care, financial costs, and non-financial costs of the service received. For each variable, multiple items were used. All variables were computed with 7-point Likert scales, where subjects provided their level of agreement to statements about their telemedicine experiences. Scales for the questionnaire items ranged from “1,” strongly disagree with the statement, to “7,” which indicated strong agreement with the statement. All items were scaled such that higher scores indicated higher levels of favorability. Means were computed from the responses to the individual items associated with a given variable (see Supplementary Material S1 for all measurement items and how variables were calculated).

Cronbach’s Alpha coefficients were calculated to demonstrate the reliability of items used in the computation of the respective variables. Mean overall satisfaction differences across each country were examined with ANOVA. Regression analysis tested the significance of the IVs as predictors of overall satisfaction. Lastly, random forest analysis, a machine learning algorithm [17], was conducted to build decision trees on different samples and take a consensus of the trees’ predictions to identify the importance of each IV for predicting overall satisfaction for each respective country.

## 3. Results

As mentioned above, a total of 1950 patients across nine countries responded to the questionnaire. Table 1 provides aggregated patient characteristics along with characteristic breakdowns within each country. In the aggregate, slightly over half (52.9%) of the patients reported that this was their first telemedicine experience. Most of the patients had received primary medical care (74.2%), and the gender breakdown was approximately equal between males (50.5%) and females (49.2%). The patients’ average age was nearly 45 years old, ranging from 20 to 89 years old. Patients were well educated with approximately 62% having attained at least a bachelor’s degree.

The items used to compute the DV and IVs were found to be reliable as shown by the Cronbach coefficients presented in Table 2. Standardized coefficients were based upon item covariances. The reliability coefficients were typically high, ranging from 0.64 to 0.97, suggesting internal reliability [18].

Mean DV values for each of the observed countries are provided in Table 3. Overall satisfaction means ranged from 5.327 to 6.472 on the 7-point scale, indicating that patients in all observed countries were generally satisfied with the telemedicine services they received. The mean telemedicine overall satisfaction level was both the highest and lowest in Asian countries (India and Japan, respectively).

ANOVA results show that *overall satisfaction* means differed significantly across the countries observed. The ANOVA test criteria and F-value results for the hypothesis of no difference in *overall satisfaction* means are shown in Table 4. The F-statistic (F(8,1941) = 19.23) indicates a significant difference in mean *overall satisfaction* across the nine countries observed (*p* < 0.001).

Based upon significant ANOVA results, Duncan’s multiple range test, which considers the studentized range distribution, was performed to identify significant differences between *overall satisfaction* means. Based upon Duncan Groupings shown in Table 5, patients from India and Brazil had significantly higher *overall satisfaction* than patients from any of the other countries. The Japanese patients had significantly lower *overall satisfaction* than the other countries. There was no significant difference in *overall satisfaction* among patients from Australia, Canada, Germany, Mexico, the U.K., and the U.S.

For the countries observed, Table 6 shows means for each of the satisfaction dimensions (IVs). Patients had the highest ratings for patient-centered care in all of the countries except for Japan, where non-financial costs was rated the highest. In addition to having the highest overall satisfaction, telemedicine patients in India and Brazil also had the highest evaluations for the patient-centered care and health benefits predictor dimensions. The lowest evaluated predictor dimension was financial costs for most countries, except for Germany, where health benefits was rated the lowest.

Multiple linear regression was used to test whether the IVs significantly predicted telemedicine overall satisfaction (DV). The results of that analysis are provided in Table 7. The fitted regression model was overall satisfaction = −0.046652 + 0.25381(health benefits) + 0.54663(patient-centered care) + 0.06363(financial costs) + 0.27348(non-financial costs). The overall regression model was statistically significant (R^2^ = 0.694, F(4, 1945) = 1100.82, *p* < 0.001). In addition, it was observed that all four IVs were significant predictors of overall satisfaction (*p*-values for each IV ≤ 0.001).

Random forest analysis was performed, using the IVs to identify the most important dimension for predicting overall satisfaction*.* This analysis considers the percent increase in Mean Square Error (MSE) due to the removal of a given IV from the model. Multiple random forest models, each consisting of 500 regression trees, were generated using different random starting seeds. The relative importance of the predictors remained consistent across models, indicating that the resulting importance rankings were stable. As shown in Table 8, patient-centered care was the most important overall satisfaction predictor for all countries except for the U.S. and Japan, where non-financial costs was a more important predictor. Health benefits was the second most important predictor in Brazil, Canada, Germany Mexico, and the U.K. The least important predictor across all nine countries was financial costs.

In addition to the country comparisons presented above, further analysis was performed to determine whether patient characteristics had a significant impact on overall satisfaction as well as the four satisfaction dimensions (health benefits, patient-centered care, financial costs, and non-financial costs). Specifically, LASSO regression analysis was used, incorporating country, age, gender, education level, type of care received, and number of experiences, as predictors of overall satisfaction and the four satisfaction dimensions. The LASSO models were selected using cross-validation. Across the demographic and contextual factors examined, country was consistently the dominant factor retained by the model, with the largest and most persistent nonzero coefficient. In contrast, the demographic characteristics generally contributed little to the model, with their coefficients frequently being reduced to or near zero. These results suggested that the inclusion of demographic characteristics would not materially alter the national differences, which account for substantially more of the observed variation in each of the satisfaction dimensions than the demographic predictors examined. However, it was observed that overall satisfaction was slightly lower for patients who were older as compared to those who were younger. Also, perceptions of non-financial costs were slightly lower for first-time telemedicine patients than for those with previous experiences.

## 4. Discussion

The first research question (R_1_) relates to whether patient overall satisfaction varies across countries. While the results in Table 3 demonstrate that telemedicine patients across all countries observed were generally satisfied with the service they received, the ANOVA results (Table 4) indicate there are some significant differences. As shown by the overall satisfaction mean groupings (see Table 5), patients in India and Brazil reported the highest overall satisfaction (Duncan Grouping A) while patients in Japan reported the lowest overall satisfaction (Duncan Grouping C).

Why are telemedicine patients in India and Brazil more satisfied with telemedicine care, and why are patients in Japan less satisfied? Data collected on the four satisfaction dimensions may provide some insights. Table 6 shows that patients in most countries evaluated the patient-centered care received (i.e., the patient’s perceptions of the provider) higher than that of the other satisfaction dimensions. Notably, India and Brazil also rated the health benefits received (i.e., the patient’s perceptions of healthcare access and health outcomes) almost as highly as patient-centered care, which was not seen in patients from the other countries. This finding may reflect the transformative role of telemedicine in expanding healthcare access to settings where traditional services had previously been limited. For example, since 2022, India has rapidly expanded 5G network access. Recent research has found that 5G’s ultra-low latency and elevated packet-delivery reliability drastically improve real-time teleconsultations [19]. Furthermore, in Brazil, the integration of innovative telemedicine platforms expanded the availability of telemedicine [7]. These advancements may have improved perceptions of health benefits, which in turn may have improved overall satisfaction.

In contrast, Japanese patients appeared to be the most critical in their perceptions of the satisfaction dimensions, with the lowest average ratings in all categories relative to other countries but for health benefits. The largest observed nominal gap between Japan and other countries was in the category of patient-centered care (Table 6). A possible explanation could be cross-cultural differences in communication style and expectations between Eastern and Western languages (i.e., high-context vs. low-context languages). Previous research shows Japanese patient interviews include less socio-emotional “small talk” than U.S. patient interviews and include more silence [20], consistent with a high-context language style that relies more on nonverbal cues—precisely the cues video interactions can dampen. If Japanese patients require face-to-face encounters to see and recognize subtle, rapport-building cues, telemedicine may feel less “caring,” negatively impacting their perceptions of patient-centered care and subsequently lowering overall satisfaction.

Table 6 also shows that patients from all countries except Germany rated financial costs as the least favorable satisfaction dimension. This observation may be the result of country-specific financial barriers to telemedicine use [4]. For example, inconsistent or confusing reimbursement policies across regions or healthcare payor systems can lead to higher out-of-pocket costs for patients, negatively impacting perceptions of financial costs.

The second research question (R_2_) addresses whether the predictive importance of the four telemedicine patient satisfaction dimensions varies across countries. The results confirm that the most important predictor of overall satisfaction varies by country (see Table 7). While predictive importance does not necessarily demonstrate a causal effect, the random forest analysis provided insights into overall satisfaction. Specifically, random forest analysis (see Table 8) found that across most countries, patient-centered care was the strongest predictor of satisfaction, underscoring the importance of healthcare providers exhibiting good interpersonal skills in virtual encounters. Exceptions were observed in Japan and the U.S., where non-financial cost*s*—such as convenience, time efficiency, and technological complexity—were more influential. In both the U.S. and Japan, it seems that patients consider timesaving to be a top priority. In the U.S., a 2022 J.D. Power survey found convenience and speed to be the top stated reasons for using telehealth (61% cite convenience, 49% faster access) [21]. In Japan, for those in the workforce, persistently long working hours heighten the opportunity cost of clinic visits, making time efficiency comparatively salient in patient valuations of telemedicine service [22]. Furthermore, with Japan’s aging society, a growing number of telemedicine users are likely to be older and technologically averse. As such, technological ease-of-use or lack thereof could have a large impact on telemedicine satisfaction. Interestingly, financial costs, which had the lowest satisfaction levels among most countries, consistently emerged as the least important predictor of overall satisfaction, signifying that the experiential aspects of telemedicine are valued above monetary considerations.

In sum, the findings highlight the need for country-specific tailored strategies to improve telemedicine satisfaction. Consistent with previous research [16], findings from this study indicate that strengthening patient-centered care remains essential in all countries, but attention to country-specific expectations is necessary for telemedicine to achieve its full potential. Medical providers in countries with lower patient satisfaction could adopt best practices from providers in countries where patient satisfaction is higher. In emerging markets such as Brazil and India, gains in rural patient access and technology infrastructure will likely continue to be critical to telemedicine advancements. In Japan, aligning telemedicine services with cultural expectations may improve acceptance. Finally, in the U.S. and Japan, streamlining user experiences through the development of user-friendly and timesaving telemedicine platforms could further enhance adoption and satisfaction.

This study has several limitations. First, the study only measured subjective perceptions of telemedicine service not objective clinical health outcomes, thereby limiting interpretations of the clinical benefits of telemedicine. Second, the cross-sectional design captures patient satisfaction at a single point in time, limiting the ability to assess changes in perceptions as telemedicine adoption evolves. Third, although translations of the survey were used in non-English-speaking countries, subtle cultural or linguistic differences may have influenced how respondents interpreted items. Fourth, the study relied on self-reported satisfaction measures, which may be subject to recall or response bias. Fifth, data was collected using convenience sampling and the majority of respondents were primary-care encounters, thus limiting the generalizability of the results to each country’s respective general populations. In addition, online panel recruitment may bias the sample toward more digitally literate patients, potentially inflating satisfaction scores. Finally, while nine countries were included, findings may not be generalizable to all healthcare contexts, particularly low-income nations with limited telemedicine infrastructure, such as those found in Africa.

Future studies could build on this work by employing longitudinal designs to track how patient satisfaction shifts with expanding telemedicine adoption and technological innovations. Qualitative approaches could also provide deeper insight into cultural expectations that shape patient perceptions. Additionally, examining provider perspectives alongside patient experiences may yield a more comprehensive understanding of telemedicine patient satisfaction. Comparative research that includes lower-income regions would further enrich global perspectives on the opportunities and challenges of telemedicine.

## 5. Conclusions

Telemedicine is expanding access to healthcare, but patient satisfaction with these services varies across national contexts. By adopting a multidimensional framework, this study shows that the predictors of satisfaction differ across countries. Patient-centered care emerged as the most influential predictor in most countries, underscoring the importance of rapport building in virtual care. In Japan and the U.S., however, non-financial factors such as convenience and technological ease were more salient, reflecting differences in local expectations.

These findings suggest that improving telemedicine satisfaction may requires a dual approach: strengthening patient-centered care globally while addressing country-specific barriers such as technological usability, reimbursement systems, and cultural preferences. A nuanced understanding of these dimensions may help providers and policymakers tailor telemedicine services more effectively.

## Figures and Tables

**Table 1:** Sample Patient Characteristics of Observed Countries.

Country	Age	Gender		Education Level *		Type of Telemedicine Care			Number of Telemedicine Experiences	
	Mean	Male	Female	Low	High	Primary	Specialty	Emergency	First Time	Multiple Times
Australia	48.8	48%	51%	58%	41%	89%	8%	3%	35%	65%
Brazil	36.9	46%	54%	37%	63%	61%	33%	6%	44%	56%
Canada	46.8	41%	59%	37%	63%	74%	13%	13%	45%	55%
Germany	44.7	59%	41%	58%	41%	65%	26%	9%	48%	52%
India	39.3	59%	41%	7%	93%	69%	24%	7%	71%	29%
Japan	53.5	62%	38%	30%	69%	87%	8%	5%	78%	22%
Mexico	40.5	44%	55%	28%	72%	78%	16%	6%	68%	32%
United Kingdom	39.2	48%	51%	36%	63%	73%	18%	9%	50%	50%
United States	52.7	47%	52%	53%	46%	73%	23%	4%	35%	65%
Overall	44.6	51%	49%	38%	62%	74%	19%	7%	53%	47%

* Low = under a Bachelor’s Degree; High = Bachelor’s Degree or higher Note: Less than or equal to 0.5 percent of patients did not report one or more of the above personal characteristics.

**Table 2:** Cronbach’s Alpha Coefficients for Measurement Items by Country.

	Health Benefits (5 Items)	Patient-Centered Care (14 Items)	Financial Costs (4 Items)	Non-Financial Costs (5 Items)	DV: Overall Satisfaction (4 Items)
Country	Standard Coefficient	Standard Coefficient	Standard Coefficient	Standard Coefficient	Standard Coefficient
Australia	0.80	0.97	0.78	0.86	0.93
Brazil	0.76	0.96	0.60	0.69	0.92
Canada	0.85	0.97	0.74	0.83	0.93
Germany	0.87	0.95	0.71	0.80	0.93
India	0.82	0.89	0.65	0.75	0.79
Japan	0.78	0.97	0.73	0.88	0.91
Mexico	0.86	0.97	0.74	0.86	0.94
United Kingdom	0.81	0.94	0.64	0.71	0.88
United States	0.83	0.97	0.66	0.88	0.95

**Table 3:** Overall Satisfaction (DV) Means and Standard Deviations (SDs) by Country.

Country	Overall Satisfaction	
	Mean	SD
India	6.472	1.25
Brazil	6.295	1.05
Mexico	5.956	1.27
United Kingdom	5.938	1.19
United States	5.833	0.64
Canada	5.814	1.11
Germany	5.767	1.19
Australia	5.744	0.99
Japan	5.327	1.33

**Table 4:** ANOVA Results.

Source	DF	Sums of Squares	Mean Square	F-Value	*p*-Value > F
Model	8	194.7576	24.3447	19.23	<0.001
Error	1941	2456.6791	1.2657		
Corrected Total	1949	2651.4367			

**Table 5:** Duncan Mean Overall Satisfaction (DV) Groupings.

Country	Overall Satisfaction	Duncan Mean Groupings *
India	6.472	A—Highest Satisfaction
Brazil	6.295	
Mexico	5.956	B—Satisfied
United Kingdom	5.938	
United States	5.833	
Canada	5.814	
Germany	5.767	
Australia	5.744	
Japan	5.327	C—Lowest Satisfaction

* Means with different letters are significantly different.

**Table 6:** Satisfaction Dimension (IVs) Means and Standard Deviations (SDs) by Country.

	Health Benefits		Patient-Centered Care		Financial Costs		Non-Financial Costs	
Country	Mean	SD	Mean	SD	Mean	SD	Mean	SD
Australia	5.23	1.01	5.754 *	0.98	4.813	1.22	5.454	1.05
Brazil	5.77	0.85	6.152 *	0.85	5.213	1.03	5.659	0.86
Canada	5.24	1.04	5.801 *	0.97	5.126	1.13	5.526	1.00
Germany	4.99	1.17	5.706 *	0.99	5.140	1.02	5.465	0.99
India	5.93	0.96	6.166 *	0.69	5.673	0.93	5.842	0.89
Japan	5.15	0.87	5.211	0.94	4.619	1.07	5.345 *	1.04
Mexico	5.29	1.09	5.834 *	1.03	5.086	1.11	5.571	1.06
United Kingdom	5.31	1.03	5.806 *	0.91	4.921	1.03	5.506	0.85
United States	5.19	1.05	5.880 *	0.98	5.181	1.01	5.534	1.10

* The most favorable (highest mean) dimension for a given country.

**Table 7:** Regression Results.

ANOVA						
	DF	SS	MS	F-Value	*p*-Value > F	
Regression	4	1839.0824	459.77059	1100.82	<0.001	
Residual	1945	0812.3543	000.41766			
Total	1949	2651.4367				
	**Coefficients**	**Standard Error**	**t-Value**	***p*** ** -Value > t**	**95% Confidence Limits**	
Intercept	−0.4665	0.09814	−4.75	<0.001	−0.659	−0.274
Health Benefits	0.2538	0.01943	13.06	<0.001	0.216	0.292
Patient-Centered Care	0.5466	0.02236	24.45	<0.001	0.503	0.590
Financial Costs	0.0636	0.01644	3.87	0.001	0.031	0.096
Non-Financial Costs	0.2734	0.02001	13.66	<0.001	0.234	0.313

Note: Root Mean Square Error = 0.646.

**Table 8:** Random Forest Analysis—Predictive Importance of Independent Variables.

Percent Improvement in Model Mean Square Error				
Country	Patient- Centered Care	Non-Financial Costs	Health Benefits	Financial Costs
Australia	26.29 *	24.07	23.21	7.24
Brazil	27.65 *	9.69	20.65	4.54
Canada	24.37 *	17.52	22.50	8.41
Germany	33.06 *	18.32	20.16	8.76
India	24.20 *	17.14	10.73	7.33
Japan	30.92	31.76 *	20.63	16.43
Mexico	26.39 *	21.34	25.96	12.47
United Kingdom	30.65 *	9.16	24.54	8.79
United States	23.47	28.15 *	19.00	16.77
**Overall**	60.11	44.64	42.80	24.11

* The most important predictor of *overall satisfaction* for a given country. Note that these results do not establish that one satisfaction dimension is more reliably important than another.

## Data Availability

The participant-level dataset is not publicly available because the informed consent provided to participants specified that study data would be reported only in aggregate form. Aggregate data supporting the findings of this study are available within the article. Requests for additional data may be directed to the corresponding author, subject to institutional and ethical requirements.

## Supplementary Materials

The following supporting information can be downloaded at https://www.mdpi.com/article/10.3390/healthcare14203493/s1: Supplementary Material S1. Questionnaire Items.
