# Supplementary material for: Telemedicine Patient Satisfaction Across Nine Countries: A Cross-Sectional Comparative Study
Source: Healthcare (Basel). 2026 Oct 17;14(20):3493. doi: 10.3390/healthcare14203493 (PMC13704427; doi:10.3390/healthcare14203493)
Supplement: Supplementary file 1 [file healthcare-14-03493-s001.zip › Supplementary Material S1. Questionnaire Items.pdf]

Supplementary Material S1: Telemedicine Patient Satisfaction Questionnaire Items

Questions about Online Medical Services – Medical services were rated on a scale of 1 to 7, where 1=Strongly Disagree; 2=Disagree; 3=Somewhat Disagree; 4=Neutral; 5=Somewhat Agree; 6=Agree; and 7=Strongly Agree.

Health Benefits (computed as a mean from 5 items)
1. Telemedicine provides greater access to healthcare specialists.
2. Telemedicine provides greater overall healthcare access.
3. Telemedicine provides safety from exposure to diseases.
4. Telemedicine provides excellent health care.
5. Telemedicine effectively improved my medical condition.

Patient-Centered Care (computed as a mean from 14 items)
1. The telemedicine healthcare providers were very knowledgeable.
2. The telemedicine healthcare providers were very polite.
3. The telemedicine healthcare providers were very credible
4. The telemedicine healthcare providers were very trustworthy.
5. The telemedicine healthcare providers accurately communicated important aspects of the service in a way I could understand.
6. The telemedicine healthcare providers were responsive to my needs.
7. I am confident that telemedicine healthcare providers will protect my privacy.
8. The telemedicine healthcare providers created an atmosphere for positive communications.
9. The telemedicine healthcare providers create an atmosphere for positive relationships.
10. The telemedicine healthcare providers were very caring.
11. The telemedicine healthcare providers were completely focused on me.
12. The telemedicine healthcare providers were very interested in my health condition.
13. The telemedicine healthcare providers were very focused on my best interests.
14. The telemedicine healthcare providers listened carefully to me.

Financial Costs (computed as a mean from 4 items)
1. Telemedicine costs less than traditional healthcare service.
2. Telemedicine does not require any additional medical insurance procedures.
3. Telemedicine services are covered by insurance.
4. Telemedicine reduces expenses.

Non-Financial Costs (computed as a mean from 5 items)
1. Telemedicine makes appointment scheduling more convenient.
2. Telemedicine decreases wait time for services.
3. Telemedicine equipment used by patients is not complex.
4. Telemedicine equipment requirements are not problematic.
5. Telemedicine technology is non-intimidating.

Overall Satisfaction (computed as a mean from 4 items)
1. I appreciate the telemedicine service I received.
2. Overall, I was satisfied with my telemedicine service.
3. In the future, I would utilize telemedicine services for my health care.
4. I will recommend telemedicine services to others.

Patient Characteristics Items

1. Is this this first time you have utilized telemedicine services?
   -Yes, this is this first time I have utilized telemedicine services.
   -No, I have utilized telemedicine services prior to this encounter.

2. Please select the category that best describes the type of telemedicine care you received:
   -Primary care
   -Specialty care
   -Emergency care

3. Please select the category that describes your gender:
   -Male
   -Female
   -Non-binary

-Prefer to not answer

4. Please enter your age as an integer:
   -Free entry textbox

5. Please select the category that describes your highest level of educations:
   -Junior high school
   -High school
   -Vocational school
   -Bachelor’s degree
   -Master’s degree
   -Doctoral degree
   -Prefer not to answer
